# Cost-Effectiveness of Minimally Invasive Tubular vs. Open Laminectomy for Pure Lumbar Spinal Stenosis: A Markov State-Transition Analysis

**DOI:** 10.1177/21925682261494612

**Published:** 2026-09-27

**Authors:** Rohan A. Phadke, Samer G. Salman, Imaad Virani, James Burnett, Maryam Y. Jawid, Arnav Kak, Alexander Mihas, Lorenzo Deveza

**Affiliations:** 1 School of Medicine, 3989 Baylor College of Medicine, Houston, TX, USA; 2 Joseph Barnhart Department of Orthopedic Surgery, 3989 Baylor College of Medicine, Houston, TX, USA

**Keywords:** stenosis, lumbar, degenerative disc disease, decompression, laminectomy, markov, economic analysis, cost effectiveness

## Abstract

**Study Design:**

Decision-analytic cost-effectiveness analysis using a Markov state-transition model.

**Introduction:**

Lumbar spinal stenosis (LSS) is the most common indication for spine surgery in older adults. Minimally invasive surgery (MIS) tubular decompression and open laminectomy produce equivalent one-year outcomes, but their long-term cost-effectiveness for pure decompression is unknown. We modeled 10-year cost-effectiveness from the U.S. payer perspective.

**Methods:**

We built a five-state Markov model for a simulated cohort of 10,000 patients aged 65 with pure LSS (no instability, deformity, spondylolisthesis, or prior surgery), using 3-month cycles, a 10-year horizon, and 3% annual discounting. QALYs used SPORT EQ-5D utilities. Perioperative complication rates came from the Nerland 2015 propensity-matched cohort (10.6% vs. 14.6%; risk difference 4.1 percentage points, 95% CI -2.6 to 10.7). Recurrence, postoperative utility, and perioperative mortality were equalized across arms. One-way, threshold, and probabilistic sensitivity analyses (PSA) were performed, including wide-uncertainty sampling of the complication difference across its confidence interval.

**Results:**

MIS yielded 5.95 QALYs at $26,283 versus 5.91 QALYs at $30,450 for open laminectomy, saving $4,167 per patient (MIS dominant). MIS was cost-effective in 81% of PSA iterations at $50,000/QALY and in 82% to 88% under wide-uncertainty sampling, and remained cost-effective at $100,000/QALY unless its complication rate exceeded 17.5%. Without any complication advantage, MIS still saved $3,454 with equal QALYs.

**Conclusion:**

For pure LSS, MIS tubular decompression was cost-effective and, in the base case, dominant over open laminectomy. The advantage reflects the complication difference and the lower index cost, and should inform rather than determine coverage policy.

## Introduction

Lumbar spinal stenosis (LSS) is the most prevalent cause of spinal surgery in adults over 65 years of age.^1^ The Spine Patient Outcomes Research Trial (SPORT) demonstrated that decompressive surgery improves pain and physical function more than nonoperative care through 4 years.^2-4^ At 8-year follow-up, the cumulative reoperation rate was 18%.^4^ This reoperation burden makes the downstream economics of the index approach important.

Minimally invasive surgery (MIS) tubular decompression has emerged as an alternative to traditional open laminectomy for LSS. MIS tubular decompression here denotes a unilateral tubular or microendoscopic approach with bilateral decompression, and open laminectomy conventional midline decompression, performed with or without microscope magnification. The Nerland et al 2015 pragmatic comparative effectiveness study, using data from the Norwegian Registry for Spine Surgery (n = 885), demonstrated equivalence in one-year Oswestry Disability Index and EQ-5D outcomes between microdecompression and open laminectomy, while reporting a lower perioperative complication rate (9.8% vs. 15.0% in the full cohort, p = 0.018; 10.6% vs. 14.6% in the propensity-matched subcohort of 492 patients, the values used in this model) and shorter hospital stays with MIS.^5^ An operating microscope was used in every microdecompression and in 39.4% of laminectomies in that cohort, so the arms differ in the extent of muscular exposure rather than in magnification. Subsequent meta-analyses have confirmed that MIS tubular decompression is associated with fewer overall complications (OR 0.42, 95% CI 0.21-0.86), with equivalent patient-reported outcomes and reoperation rates.^6,7^

Despite equivalent clinical effectiveness, no Markov state-transition model has compared the long-term cost-effectiveness of MIS tubular decompression versus open laminectomy for pure LSS without spondylolisthesis. Prior studies compared surgery with nonoperative care or decompression with versus without fusion, leaving a gap between surgical techniques.^8-10^ We therefore constructed a Markov model to compare the 10-year cost-effectiveness of MIS tubular decompression versus open laminectomy from the U.S. healthcare payer perspective.

## Methods

### Model structure and cohort

We constructed a five-state Markov state-transition model (Figure 1) comparing minimally invasive surgery (MIS) tubular decompression with open laminectomy for pure lumbar spinal stenosis (LSS) without instability or spondylolisthesis. In brief, the model follows a group of identical patients through 40 three-month cycles; in each cycle a patient either remains in the current health state or moves to another with a fixed probability, accruing that state’s cost and quality-of-life value. Pure LSS was defined as in the source cohort^5^: symptomatic central or lateral-recess stenosis at one or two adjacent levels with neurogenic claudication or radiculopathy refractory to non-operative care, excluding dynamic instability, any degenerative or isthmic spondylolisthesis, coronal or sagittal deformity, prior surgery at the index level, and infection, tumor or fracture, each of which independently raises cost and reoperation risk. A hypothetical cohort of 10,000 patients aged 65 entered an arm-specific Postoperative state for one 3-month cycle, then transitioned among Well, Symptomatic, Post-revision, and Death. The cohort is simulated rather than an actual patient sample: hypothetical patients are redistributed across the health states each cycle according to the transition probabilities in Table 1. Cohort size scales event counts only and does not affect per-patient costs, QALYs, or the ICER. We used 3-month cycles, a 10-year horizon, half-cycle correction, and a 3% annual discount rate, taking the U.S. healthcare payer perspective. Reporting followed the Consolidated Health Economic Evaluation Reporting Standards (CHEERS) 2022.^11^

**Figure 1.**
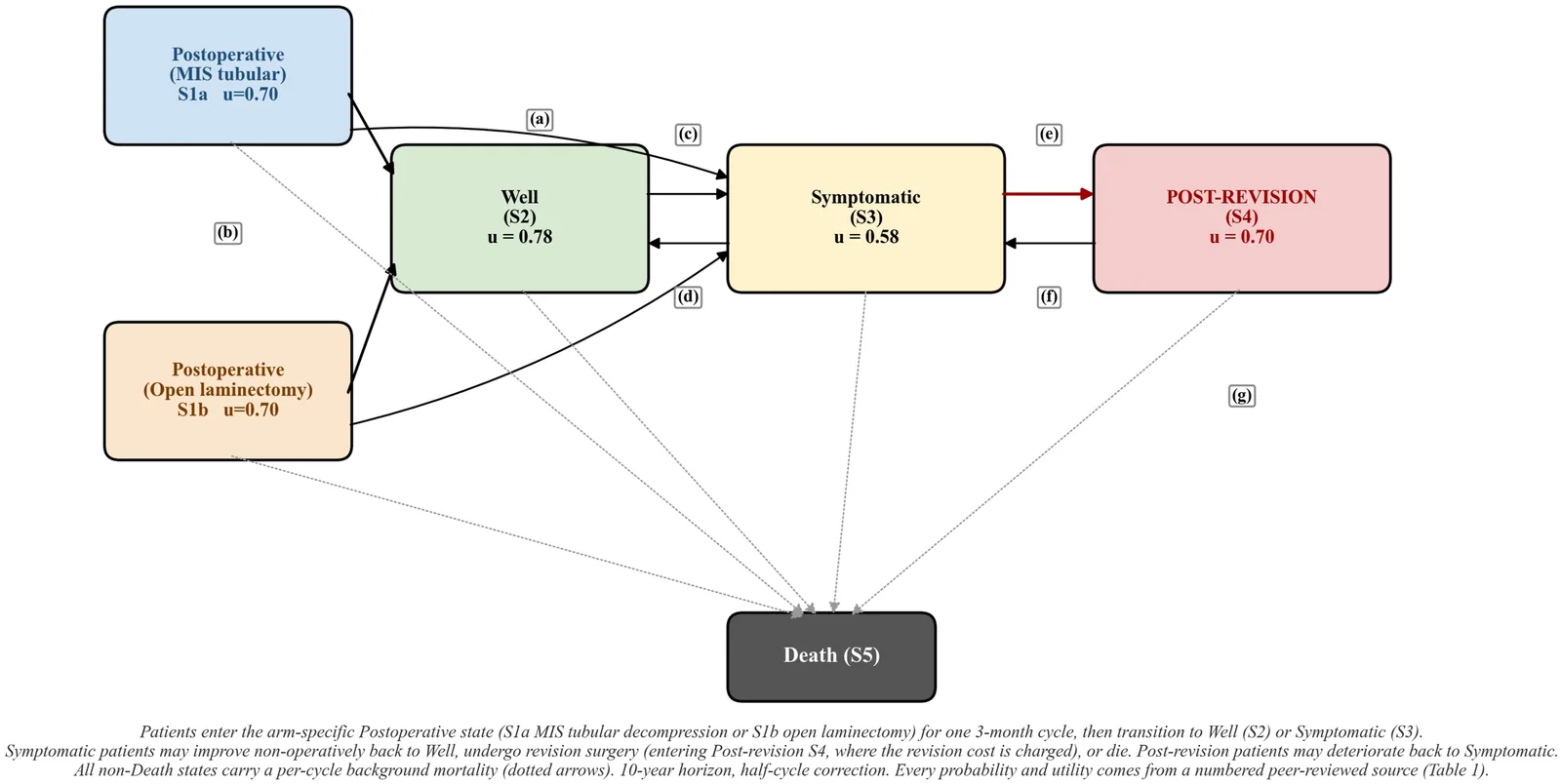
Five-state Markov model structure for the decompression-only comparison. Patients enter the arm-specific Postoperative state (S1a MIS tubular decompression or S1b open laminectomy) for one 3-month cycle, then transition to Well (S2) or Symptomatic (S3). Symptomatic patients may improve non-operatively back to Well, undergo revision surgery (entering Post-revision S4, where the weighted revision cost is charged), or die. Post-revision patients may deteriorate back to Symptomatic. Reoperation is tracked as an explicit state because it is the principal high-cost adverse event distinguishing MIS from Open. Arrow key: (a) perioperative complication rate: MIS 10.6%, open laminectomy 14.6% (Nerland 2015, propensity-matched); (b) 3-month postoperative tunnel state; utility equalized across both arms (see Methods); (c) recurrence from Well to Symptomatic: 1.7%/yr, equalized across both arms; (d) non-operative improvement from Symptomatic to Well: 20%/yr; (e) revision surgery from Symptomatic to Post-revision: 40%/yr, weighted revision cost charged on entry to S4; (f) deterioration from Post-revision back to Symptomatic: 4%/yr; (g) reoperation composition: 60% repeat decompression, 40% salvage fusion

**Table 1.** Markov model input parameters for the decompression-only comparison. Evidence tier is shown with each source: DIRECT (exact published value), CALIBRATED (set to hit a specific empirical target), EQUALIZED (held identical across both arms), DERIVED (arithmetic from a primary source), or JUDGMENT (acknowledged estimate, swept in sensitivity analysis). PSA dist. = probabilistic sensitivity analysis distribution

Parameter	Base value	Evidence tier + Source(s)	PSA dist.
Costs (2023 USD, payer perspective)	​	​	Gamma
MIS index surgery (tubular/microendoscopic decompression)	$13,500	[DERIVED] Lubelski 2014^12^: $2,311 cost difference reported for MAS vs open multilevel hemilaminectomy (not significant); absolute level assumed	Beta
Open index surgery (open laminectomy ± medial facetectomy)	$15,500	[DERIVED] Lubelski 2014^12^; see MIS index surgery. Only the between-arm difference affects the ICER	Beta
Revision surgery cost, MIS arm	$21,000	[DERIVED] Adogwa 2012^13^ prints a mean revision surgical cost of $21,060	Gamma
Revision surgery cost, Open arm	$23,500	[DERIVED] Adogwa 2012^13^; Lubelski^12^	Gamma
Annual cost - Well state	$300	[DERIVED] CMS Physician Fee Schedule 2023: CPT 99213 + 72100 + amortized 72148. Equalized with the Post-revision state; doubling it moves the incremental cost by $18	Gamma
Annual cost - Symptomatic state	$2,000	[JUDGMENT] Ongoing non-operative care. Tosteson 2008^12^ reports $7,161 direct cost/2 yr in the SPORT non-operative arm ($3,580/yr); the lower value used here is conservative, since the open arm spends more time in this state	Gamma
Annual cost - Post-revision state	$300	[EQUALIZED] CMS Fee Schedule (same as Well)	Gamma
Transition probabilities (annual unless noted)			
90-day perioperative complication, MIS	0.106	[DIRECT] Nerland 2015^5^ propensity-matched (n=492)	Beta
90-day perioperative complication, Open	0.146	[DIRECT] Nerland 2015^5^ propensity-matched (n=492)	Beta
Dural tear rate, MIS (informational)	2.3%	[DIRECT] Nerland 2015^5^	Beta
Dural tear rate, Open (informational)	5.1%	[DIRECT] Nerland 2015^5^; Lurie 2015^4^ (SPORT overall 9%)	Beta
Perioperative mortality, BOTH ARMS	0.00081	[EQUALIZED] Poorman 2018^14^ NIS, decompression-only	Beta
Well → Symptomatic recurrence, BOTH ARMS	0.017	[CALIBRATED+EQUALIZED] Lurie 2015^4^ SPORT 8-yr (18%); Nerland^5^; Phan^7^	Beta
Symptomatic → Post-revision (revision surgery)	0.40	[CALIBRATED] Lurie 2015^4^ SPORT 8-yr 18% cumulative	Beta
Symptomatic → Well (non-op improvement)	0.15	[JUDGMENT] No source reports this transition rate; equalized across arms and swept in OWSA and PSA	Beta
Post-revision → Symptomatic (deterioration)	0.04	[JUDGMENT] Adogwa 2012^13^ revision-failure cohort	Beta
Revision perioperative mortality	0.00081	[EQUALIZED] Poorman 2018^14^ NIS	Beta
Background all-cause mortality (age 65–75)	0.022	[DIRECT] Arias 2023^15^ CDC U.S. Life Tables 2021	Beta
Health-state utilities (EQ-5D index)			
Postoperative utility, BOTH ARMS (1st 3 mo)	0.70	[EQUALIZED] McAnany 2014^10^ decrement method. Provably non-influential: identical in both arms, so the incremental result is unchanged for any value	Beta
Well	0.78	[DERIVED] Tosteson 2008^16^ Table 2: 1.54 discounted QALYs over 2 yr, stenosis surgical arm; 1.54/(1 + 1/1.03) = 0.78	Beta
Symptomatic	0.58	[DIRECT] Tosteson 2008^16^ Table 1: “EuroQol EQ-5D U.S. health state value 0.58 (0.2)”, baseline, stenosis surgical cohort	Beta
Post-revision	0.60	[DIRECT] Adogwa 2012^13^: EQ-5D “improved from a preoperative value of 0.18 to 0.60″ over 2 years	Beta
Return-to-work (secondary outcome)			
RTW at 6 weeks, MIS	75%	[JUDGMENT] Secondary outcome; anchored to the Nerland 2015^5^ length-of-stay advantage but not reported as a return-to-work rate	Beta
RTW at 6 weeks, Open	50%	[JUDGMENT] Secondary outcome; see RTW at 6 weeks, MIS	Beta
RTW at 3 months, MIS	92%	[JUDGMENT] Secondary outcome; see RTW at 6 weeks, MIS	Beta
RTW at 3 months, Open	78%	[JUDGMENT] Secondary outcome; see RTW at 6 weeks, MIS	Beta
Median daily wage (2023 USD)	$229	[DERIVED] U.S. BLS Q4 2023^17^: median usual weekly earnings $1,145/5 workdays	Gamma
Workdays lost/yr (Symptomatic state)	15	[JUDGMENT] Societal scenario only; not reproducible from Tosteson 2008^16^ productivity data, which imply ∼3.5 days/yr averaged over a largely retired cohort	Beta

### Health states and transitions

After the index procedure, patients reached either the Well state (asymptomatic, durable decompression) or the Symptomatic state (persistent or recurrent stenotic symptoms). These states are analytic constructs, not categories reported by the source studies: Well corresponds to durable relief after decompression (EQ-5D 0.78) and Symptomatic to persistent or recurrent claudication at the pre-treatment level (0.58). Entry into each state was governed by the perioperative complication rate and then calibrated to the observed 8-year reoperation rate. Well patients could later become Symptomatic through recurrence, and Symptomatic patients could improve back to Well with non-operative care or progress to revision surgery. Revision moved a patient into the Post-revision state. Every non-Death state carried a per-cycle background mortality risk, and Death was an absorbing state. The two arms differed at only three inputs, index cost, revision cost, and the 90-day complication rate; every transition probability beyond the index procedure was shared, by design (see Input parameters).

### Input parameters

All inputs and their evidence tiers are listed in Table 1. Inputs followed a prespecified hierarchy: the one comparative input separating the arms came from the best available head-to-head source rather than a pooled estimate, and all other inputs from the largest or most representative source for that parameter. The arms differ at only three inputs, so between-study heterogeneity in the shared parameters cannot by itself create a difference between them; a meta-analytic alternative to the complication estimate is tested in the scenarios. Perioperative (90-day) complication rates were taken directly from the Nerland 2015^5^ propensity-matched cohort (10.6% for MIS vs. 14.6% for open laminectomy). Health-state utilities were EuroQol-5 Dimension (EQ-5D) index values from the Spine Patient Outcomes Research Trial (SPORT).^16^ Index procedure costs (Lubelski 2014,^12^ inflated to 2023 U.S. dollars) were $13,500 for MIS and $15,500 for open laminectomy. Revision surgery occurred at a rate of 40% per year from the Symptomatic state, charged at a weighted cost reflecting 60% repeat decompression and 40% salvage fusion (Adogwa 2012^13^). Background mortality came from CDC life tables^15^ and perioperative mortality from the National Inpatient Sample.^14^ Three long-term parameters, the recurrence rate (1.7% per year), postoperative utility (0.70), and perioperative mortality, were deliberately held equal across the two arms because no decompression-specific study supports an arm-level long-term difference. Nerland 2015^5^ found equivalent 1-year Oswestry Disability Index and EQ-5D scores and the pooled analyses found no significant difference in reoperation,^6,7^ so assigning MIS a long-term advantage would be extrapolation; each equalization was relaxed against MIS in sensitivity analysis. Recurrence and revision rates were calibrated so that the model reproduced the 18% cumulative 8-year reoperation rate observed in SPORT (Lurie 2015^4^).

### Analyses

We computed the incremental cost-effectiveness ratio (ICER) and the incremental net monetary benefit (iNMB) at willingness-to-pay (WTP) thresholds of $50,000, $100,000, and $150,000 per quality-adjusted life-year (QALY). QALYs were the time spent in each health state multiplied by that state’s EQ-5D index utility, summed across cycles with half-cycle correction and discounting; no disability or functional score was converted into utility, and Oswestry Disability Index data were used only to establish clinical equivalence between the approaches. Uncertainty was assessed by one-way sensitivity analysis (OWSA) on every parameter and by a 10,000-iteration probabilistic sensitivity analysis (PSA) using Beta distributions for probabilities and utilities and Gamma distributions for costs. Seven prespecified scenarios and subgroup analyses were performed (Table 5). The complication difference was not statistically significant in the matched cohort (36 of 246 vs. 26 of 246 patients; risk difference 4.1 percentage points, P = 0.23 by McNemar’s test), so we recovered its standard error from the published P value (3.4 percentage points; 95% CI -2.6 to 10.7) and added a threshold analysis; a wide-uncertainty PSA drawing the risk difference from that distribution; and scenarios that relaxed each equalized parameter against MIS, removed the complication advantage, equalized index and revision costs, substituted the meta-analytic odds ratio (0.42), and modeled learning-curve complication rates 25% and 50% above base case (Table 5).

### Model validation

Internal validity was confirmed by verifying that transition probabilities summed to 1.0 in every cycle. For external calibration, the model reproduced 8-year cumulative reoperation rates of 15.0% for MIS and 18.0% for open laminectomy, the latter matching the 18% pooled rate reported in SPORT, whose surgical arm was open laminectomy (Lurie 2015^4^).

## Results

### Base case

MIS was the dominant strategy, both less costly and more effective (Table 2). MIS produced 5.950 discounted QALYs at $26,283 per patient, versus 5.909 QALYs at $30,450 for open laminectomy, a saving of $4,167 per patient with a net gain of 0.041 QALYs. The lower MIS index and revision costs accounted for $3,454 of the saving and the complication difference for the remaining $713.

**Table 2.** Base-case cost-effectiveness results for MIS tubular decompression vs. open laminectomy, 10-year horizon, 3% annual discount rate, U.S. payer perspective

Strategy	Discounted cost (USD)	Discounted QALYs	Incremental cost (USD)	Incremental QALY	ICER (USD/QALY)
Open laminectomy	$30,450	5.909	-	-	Reference
MIS tubular decompression	$26,283	5.950	$-4,167	+0.041	MIS dominant
Undiscounted total (MIS)	$27,139	6.811	-	-	-
Undiscounted total (Open)	$31,410	6.764	-	-	-

### Clinical events

Over 10 years per 10,000 patients (Table 3), MIS produced 308 fewer reoperations (1,744 vs. 2,052) and 400 fewer perioperative complications (1,060 vs. 1,460). All-cause mortality was identical (1,913 deaths in each arm), reflecting equalized mortality inputs. The complication difference reduced conversion into the high-cost revision pathway and accounted for the entire QALY difference between arms (Figure 2). Return to work was analyzed only as a secondary outcome in the working-age subgroup and is reported below.

**Table 3.** Clinical events accrued per 10,000-patient cohort over the 10-year time horizon. Return to work is a secondary outcome that applies to the working-age subgroup only; the base-case cohort was aged 65

Outcome (per 10,000 cohort over 10 years)	MIS arm	Open arm	Absolute difference (MIS − Open)
Perioperative (90-day) complications	1060	1460	-400
Reoperations (entries into Post-revision)	1,744	2,052	-308
10-year all-cause deaths	1,913	1,913	-0
**Secondary outcome (working-age subgroup)**			
Patients returned to work at 6 weeks	7,500	5,000	+2,500
Patients returned to work at 3 months	9,200	7,800	+1,400

**Figure 2.**
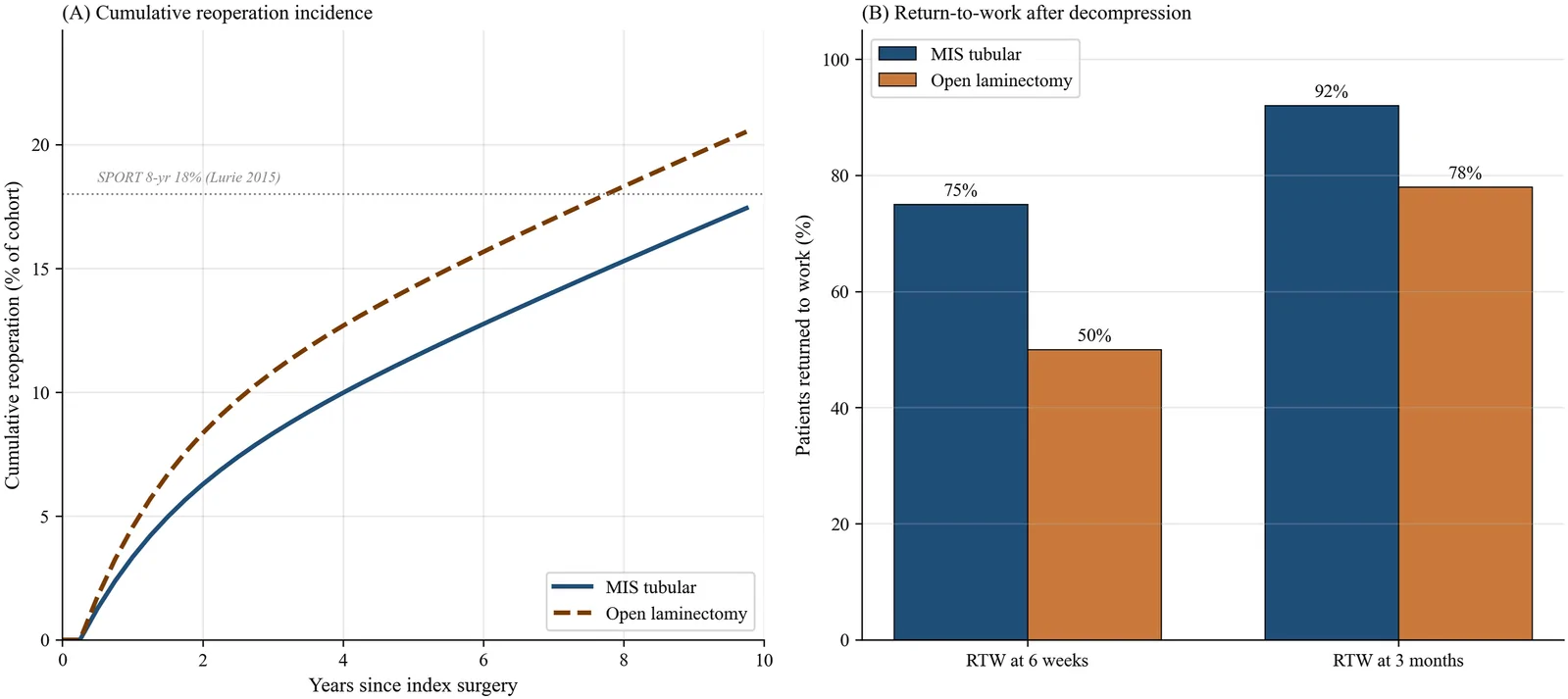
(A) Cumulative incidence of reoperation over 10 years for each strategy. The dotted reference line shows the 18% empirical 8-year reoperation rate from the SPORT trial (Lurie 2015^4^). (B) Return-to-work (RTW) at 6 weeks and 3 months by surgical approach, derived from the Nerland 2015^5^ BMJ propensity-matched cohort. RTW is a secondary outcome that applies to the working-age subgroup only; the base-case cohort was aged 65

#### Sensitivity analysis

On OWSA, the perioperative complication rate was the largest driver of value, and MIS remained favored across the plausible range of every parameter tested (Figure 3). In the PSA, MIS was cost-effective in 81.2% of 10,000 iterations at $50,000/QALY and in 83.4% at $100,000/QALY (Table 4). MIS was both less costly and more effective in 61.2% of iterations and cost-saving in 74.5%. The cost-effectiveness plane is shown in Figure 4 and the acceptability curve in Figure 5. The mean incremental net monetary benefit was positive at all three thresholds, although its 95% CI crossed zero (Table 4).

**Figure 3.**
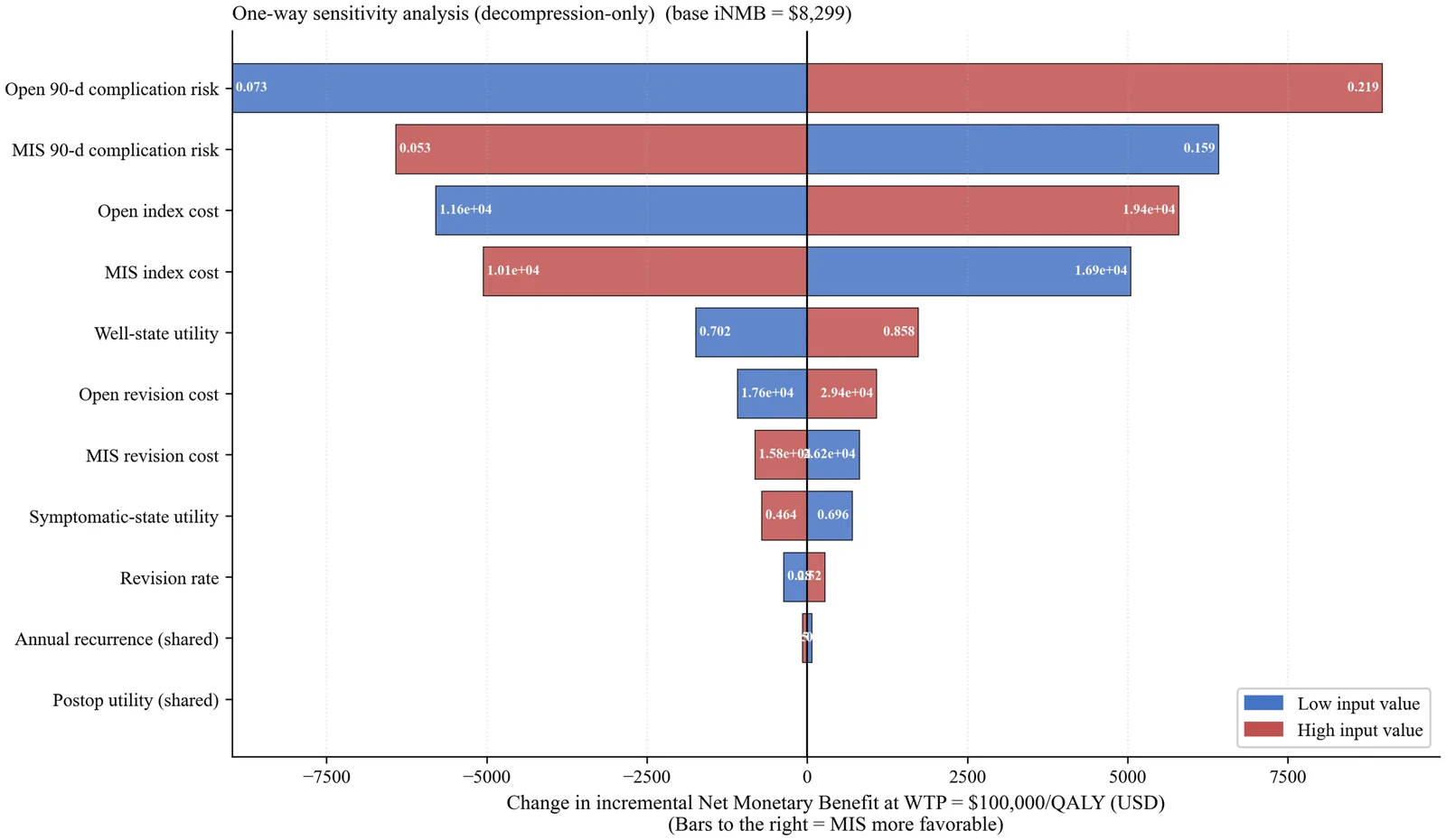
One-way sensitivity analysis (tornado plot) on the incremental Net Monetary Benefit (iNMB) at willingness-to-pay (WTP) = $100,000/QALY. Each bar shows the iNMB shift when the labeled input is varied across its plausible range

**Table 4.** Probabilistic sensitivity analysis: probability that each strategy is cost-effective and incremental Net Monetary Benefit (iNMB) across willingness-to-pay (WTP) thresholds. CrI = credible interval. The lower block reports the wide-uncertainty PSA, in which the perioperative complication risk difference was drawn from its full sampling distribution (mean 4.1 percentage points; standard error 3.4 percentage points, recovered from the P value published for the matched cohort), allowing iterations in which MIS performs worse than open laminectomy

Willingness-to-pay threshold	P(MIS cost-effective)	P(Open cost-effective)	Mean incremental NMB (USD)	95% CrI for iNMB (USD)
$50,000/QALY	81.2%	18.8%	$6,240	$-7,663 to $20,598
$100,000/QALY	83.4%	16.6%	$8,308	$-8,223 to $27,144
$150,000/QALY	84.4%	15.6%	$10,376	$-9,555 to $34,316
Mean across 10,000 PSA iterations	dC = $-4,173	dQ = +0.041	95% CrI dC: $-16,655 to $8,120	95% CrI dQ: -0.053 to +0.165
**Wide-uncertainty PSA (complication risk difference sampled across its full distribution)**				
$50,000/QALY	82.3%	17.7%	$6,158	$-7,031 to $19,401
$100,000/QALY	86.0%	14.0%	$8,233	$-6,564 to $24,048
$150,000/QALY	87.7%	12.3%	$10,308	$-6,848 to $29,412

**Figure 4.**
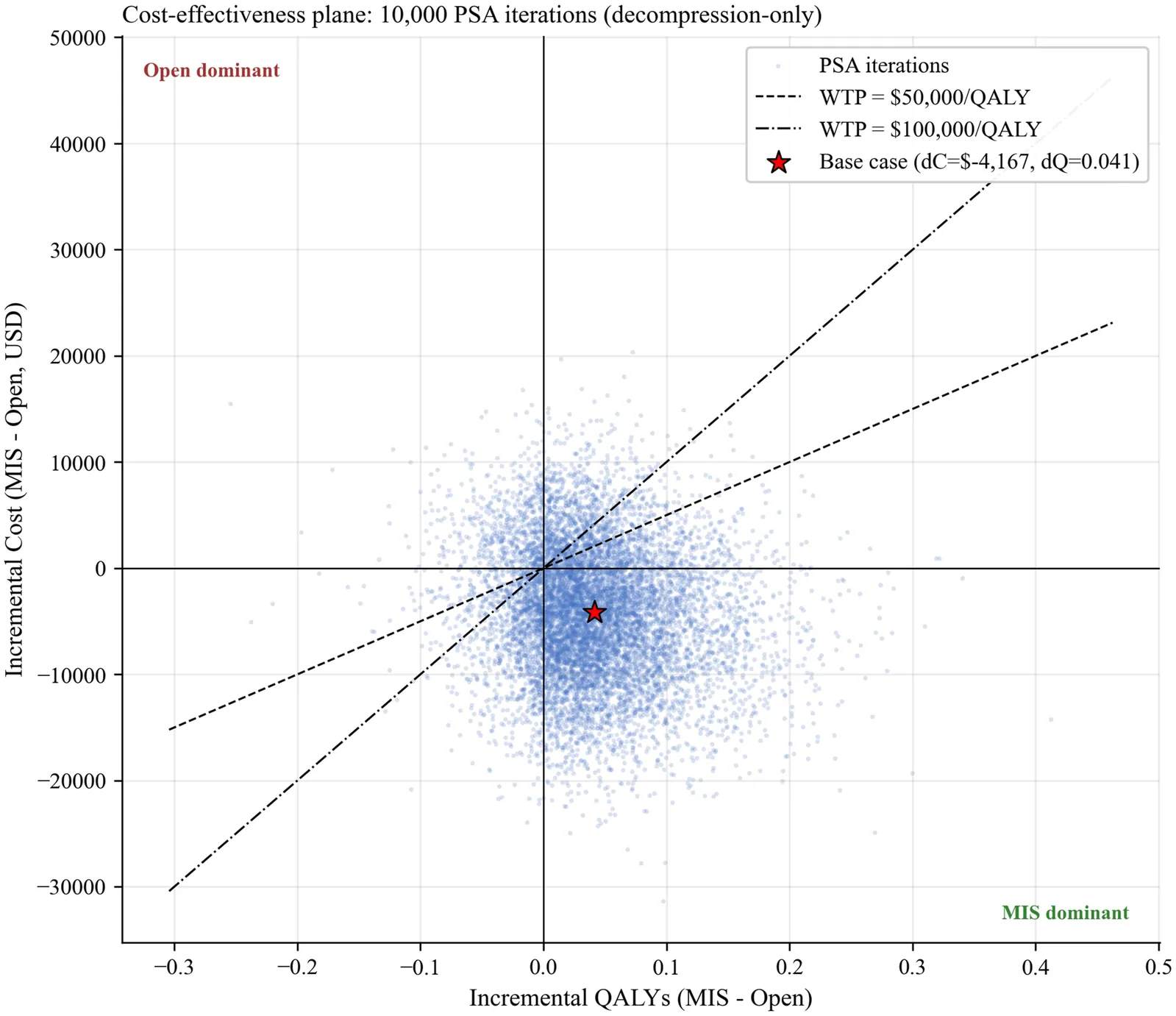
Cost-effectiveness plane. Each blue point represents one of 10,000 probabilistic sensitivity analysis (PSA) iterations. The red star marks the deterministic base case. WTP threshold lines of $50,000 and $100,000/QALY are shown

**Figure 5.**
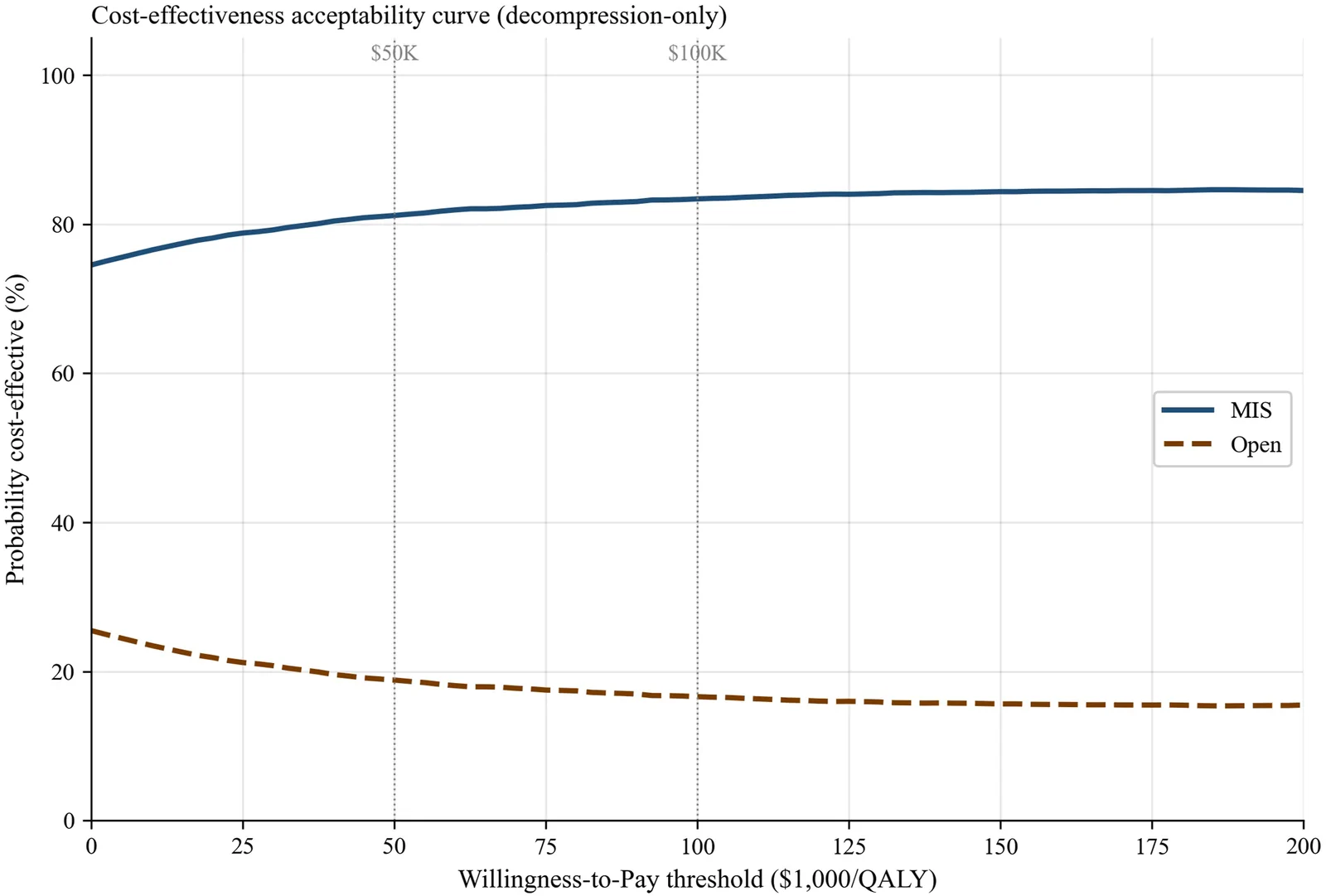
Cost-effectiveness acceptability curve (CEAC) for the decompression-only comparison across WTP thresholds from $0 to $200,000/QALY

### Scenario and subgroup analyses

MIS remained dominant in all seven prespecified analyses (Table 5): single-level and two-level disease, older (age 75) and working-age (age 55) patients, the societal perspective, a 5-year horizon, and a 50% higher reoperation rate.

**Table 5.** Subgroup and scenario analyses (decompression-only). The lower block varies the perioperative complication difference across and beyond its 95% confidence interval and relaxes each equalized parameter against MIS. †In these scenarios MIS is less costly but marginally less effective; the value shown is the cost per QALY that would have to be paid to adopt open laminectomy, far above conventional willingness-to-pay thresholds, so MIS remains the cost-effective strategy

Scenario/subgroup	Incremental cost (USD)	Incremental QALY	ICER (USD/QALY)	Conclusion
Single-level subgroup	$-3,393	+0.042	Dominant	MIS dominant
Two-level subgroup	$-4,198	+0.041	Dominant	MIS dominant
Older patient subgroup (age 75)	$-4,124	+0.037	Dominant	MIS dominant
Working-age subgroup (age 55)	$-4,208	+0.044	Dominant	MIS dominant
Societal perspective	$-11,546	+0.041	Dominant	MIS dominant
5-year horizon	$-3,978	+0.025	Dominant	MIS dominant
Higher reoperation (+50%)	$-4,400	+0.043	Dominant	MIS dominant
**Uncertainty and stress-test analyses**				
Complication risk difference at lower 95% CI limit (MIS 17.2% vs. open 14.6%)	$-2,998	-0.026	Open $113,431†	MIS cost-effective, not dominant
Complication risk difference at upper 95% CI limit (MIS 3.9%)	$-5,359	+0.110	Dominant	MIS dominant
Equal index cost in both arms	$-1,174	+0.041	Dominant	MIS dominant
Equal index and revision costs in both arms	$-749	+0.041	Dominant	MIS dominant
No complication advantage (both arms 14.6%)	$-3,454	0.000	Cost-saving, equal QALYs	MIS cost-saving
Learning curve: MIS complication +25% (13.2%)	$-3,694	+0.014	Dominant	MIS dominant
Learning curve: MIS complication +50% (15.9%)	$-3,222	-0.013	Open $239,928†	MIS cost-effective, not dominant
MIS recurrence 25% higher than open (2.1%/yr)	$-3,749	+0.024	Dominant	MIS dominant
MIS recurrence 50% higher than open (2.6%/yr)	$-3,340	+0.006	Dominant	MIS dominant
MIS 3-month postoperative utility 0.03 lower than open	$-4,167	+0.030	Dominant	MIS dominant
MIS perioperative mortality doubled vs. open	$-4,170	+0.037	Dominant	MIS dominant
Meta-analytic complication OR 0.42 applied to open rate (MIS 6.7%)	$-4,862	+0.082	Dominant	MIS dominant

### Robustness of the complication difference

The complication difference was not statistically significant in the matched cohort and was therefore varied widely (Table 5). At the unfavorable 95% limit, where MIS carries a higher complication rate than open laminectomy (17.2% vs. 14.6%), MIS lost 0.026 QALYs but still saved $2,998, with iNMB $1,676 at $50,000/QALY but only $355 at $100,000/QALY; the learning-curve scenario at 15.9% behaved similarly. Removing the complication advantage entirely equalized QALYs while MIS still saved $3,454. MIS remained cost-effective at $100,000/QALY until its complication rate reached 17.5%, and at $50,000/QALY until 19.6%. With index costs equalized across arms MIS remained dominant, saving $1,174, as it did when revision costs were also equalized ($749). Relaxing each equalized parameter against MIS left MIS dominant in every case. In the wide-uncertainty PSA, MIS was cost-effective in 82.3% of iterations at $50,000/QALY and 86.0% at $100,000/QALY. Substituting the meta-analytic odds ratio (0.42) made MIS more favorable ($4,862 saved, +0.082 QALYs), so the base case is conservative relative to pooled evidence.

## Discussion

This analysis indicates that MIS tubular decompression was the dominant strategy in the base case, producing both lower costs ($4,167 savings per patient) and higher quality-adjusted life-years (0.041 QALY gain) over a 10-year horizon. MIS was cost-effective in approximately 81% to 86% of PSA iterations, including when the complication difference was sampled across its full confidence interval, and was dominant in all seven prespecified scenario and subgroup analyses.

The QALY advantage of MIS was driven entirely by the 4-percentage-point reduction in perioperative complication rates (10.6% vs. 14.6%), derived from the Nerland et al propensity-matched cohort;^5^ the cost advantage arose mainly from the lower index and revision costs in the MIS arm. This difference was not statistically significant in the matched analysis, so the conclusion is conditional on it; the threshold and wide-uncertainty analyses indicate that the direction of the result is stable while its magnitude is not. A meta-analysis of 2,860 patients confirmed that tubular decompression is associated with significantly fewer overall complications and surgical site infections compared with open laminectomy, with no difference in reoperation rates or patient-reported outcomes.^6^ The Phan and Mobbs meta-analysis similarly reported lower reoperation rates with MIS, though not when restricted to randomized evidence.^7^ A matched cohort of 31,000 patients found lower 90-day reoperation and cerebrospinal fluid-related complications with endoscopic decompression, though higher rates of several medical complications.^18^

The model was deliberately conservative in its design. Three key long-term parameters, recurrence rate (1.7%/year), postoperative utility (0.70), and perioperative mortality (0.081%), were equalized across both arms because no decompression-specific study supports an arm-level long-term difference. A multicenter cohort in which functional gains after minimally invasive decompression attenuated over 12 months supports the recurrence pathway.^19^ Each equalization was relaxed against MIS in sensitivity analysis and MIS remained cost-saving throughout, so the conclusion does not depend on these assumptions; because the equalizations withhold from MIS any long-term benefit of tissue preservation, they bias the QALY comparison against MIS.

Index procedure costs ($13,500 MIS vs. $15,500 open, inflated to 2023 USD) were derived from the Lubelski et al systematic review,^12^ which reported a $2,311 cost difference favoring the minimal-access approach for multilevel hemilaminectomy but concluded that minimal-access and open lumbar approaches did not differ significantly in cost. Only the between-arm difference affects the ICER; equalizing index costs left MIS dominant ($1,174 saved), as did equalizing revision costs ($749). Health-state utilities came from the SPORT cost-effectiveness analysis by Tosteson et al.^16^ The post-revision utility of 0.60 is the 2-year value Adogwa et al report directly.^13^

Return to work was examined only as a secondary outcome in the working-age subgroup, because the base-case cohort was aged 65, when most patients are at or near retirement. The return-to-work advantage of MIS (75% vs. 50% at 6 weeks, 92% vs. 78% at 3 months) was derived from length-of-stay and recovery data rather than from directly observed employment outcomes, and applies to the age 55 subgroup, in whom MIS also remained dominant (Table 5). Under the societal perspective, which incorporates lost productivity, the per-patient saving increased to $11,546, a figure that applies to employed patients and not to the modeled cohort as a whole.

Several limitations should be acknowledged. First, the perioperative complication rates were drawn from a single propensity-matched observational cohort rather than a randomized controlled trial.^5^ While this represents the highest-quality comparative data available for this specific comparison, unmeasured confounding cannot be excluded. Although the unmatched complication difference was statistically significant (9.8% vs. 15.0%, p = 0.018), the propensity-matched difference used in the model (10.6% vs. 14.6%) did not reach significance (p = 0.23); its uncertainty was propagated through the probabilistic and threshold analyses, which show the direction of the result is stable across its full confidence interval even though the magnitude is not. Second, the model assumed a homogeneous cohort of 65-year-old patients with pure LSS; real-world populations are heterogeneous in comorbidity burden, stenosis severity, and surgical anatomy. A substantial proportion of surgically treated stenosis patients have concomitant degenerative spondylolisthesis and therefore fall outside the modeled population entirely. Third, the model did not incorporate the MIS learning curve or center-level volume effects; we approximated these by raising the MIS complication rate 25% and 50% above base case, and MIS remained cost-effective in both, but a surgeon or center whose complication rate approaches the 17.5% threshold would not realize the modeled benefit. Fourth, costs were modeled from the U.S. payer perspective and may not be directly transferable to other healthcare systems. Fifth, the 10-year horizon may not capture the full lifetime economic impact, particularly for younger patients.^20^ Sixth, the model is specific to decompression for pure stenosis and does not extend to other minimally invasive procedures.^21^ Seventh, the health states are analytic constructs that no source study measured directly, so state occupancy rests on calibration rather than observed data. Eighth, inputs came from different studies, so absolute estimates inherit that heterogeneity even though the arms differ at only three inputs. Finally, the model applied fixed cohort-level inputs rather than individualized perioperative risk; incorporating spine surgery risk-prediction tools could enable more personalized cost-effectiveness estimates in future work.^22^

## Conclusion

For patients with pure lumbar spinal stenosis, MIS tubular decompression offers equivalent long-term outcomes with a lower perioperative complication profile, fewer reoperations, and lower costs over a decade. Because MIS was less costly and no less effective in every analysis performed, it is a reasonable default where the necessary expertise and equipment exist. A decision-analytic model cannot by itself establish coverage policy, however: the QALY difference is small, its credible interval crosses zero, and the key comparative input is a single non-randomized estimate. These findings should therefore inform, rather than determine, payer and shared decision-making discussions. Future studies should validate these findings with data from randomized controlled trials with long-term follow-up and should incorporate patient-level heterogeneity through microsimulation modeling.
